# Case Report: Response of cutaneous lupus lesions in SLE to interferon receptor blockade parallels reduction of interferon score in blood

**DOI:** 10.3389/fimmu.2023.1253279

**Published:** 2023-09-21

**Authors:** Claudia Günther, Christine Wolf, Louisa Fennen, Sarah Rösing, Stefan Beissert, Martin Aringer, Min Ae Lee-Kirsch

**Affiliations:** ^1^ Department of Dermatology, University Hospital, Medizinische Fakultät Carl Gustav Carus, TU Dresden, Dresden, Germany; ^2^ Department of Pediatrics, University Hospital, Medizinische Fakultät Carl Gustav Carus, TU Dresden, Dresden, Germany; ^3^ Division of Rheumatology, Department of Medicine III, University Medical Center Hospital TU Dresden, Dresden, Germany

**Keywords:** interferon, IFN score, cutaneous lupus, anifrolumab, CLASI

## Abstract

Cutaneous lupus erythematosus (CLE), the main manifestation of systemic lupus erythematosus (SLE), is driven by type I interferons (IFNs) and often only partially responds to conventional therapies. Treatment of seven SLE patients with the monoclonal antibody anifrolumab induced fast and sustained remission of previously refractory CLE lesions, beginning within the first weeks of treatment. Decline in CLASI-A score was paralleled by a reduction in IFN score determined by mRNA expression of seven IFN-stimulated genes (ISGs) in blood. These data suggest that a subset of ISGs could be a valuable biomarker in CLE.

## Introduction

Cutaneous lupus erythematosus (CLE) has a wide spectrum of clinical manifestations, which demonstrate a limited response to conventional therapies. Among other cytokines, type I interferons (IFNs) have been highly implicated in the pathogenesis of CLE. Type I IFN-stimulated genes (ISGs) and proteins are typically upregulated in both the skin and blood of lupus patients. The IgG1K monoclonal antibody anifrolumab inhibits binding of all type I IFN subtypes to their single common receptor and has been EMA- and FDA-approved for the treatment of systemic lupus erythematosus (SLE). A phase 3 clinical trial for anifrolumab demonstrated improvement of CLE symptoms. Among patients with CLE Disease Area and Severity Index (CLASI) ≥10 at baseline, a reduction of 50% or more in the CLASI at week 12 occurred in 49% of the patients receiving anifrolumab compared to 25% in the placebo arm (1). However, the details of the patients’ CLE including their subtypes have not been reported.

## Methods

Here, we evaluated the clinical efficacy of anifrolumab therapy in CLE associated with SLE. In all seven patients, cutaneous lesions were refractory to previous treatment. Anifrolumab was given at a dose of 300 mg intravenously every 4 weeks, while baseline therapy with hydroxychloroquine was maintained. Two patients additionally continued methotrexate and one continued mycophenolate mofetil. In two patients, prior to anifrolumab treatment, belimumab was terminated due to insufficient control of the cutaneous manifestations (**Table 1**).

**Table 1:** Detailed patient characteristics.

No.	Age	Sex	CLE sub-type	Previous and baseline medication	Prednisolone before in mg/day	Prednisolone during in mg/day	ANA
1	67	F	DLE	HCQ, stopped: MMF			1:640
2	47	F	SCLE	HCQ, MTX, stopped: belimumab			1:640
3	60	M	SCLE	HCQ			1:640
4	26	F	ACLE + mucosal lesions	HCQ, MMF, stopped: belimumab	20	5	1:5,120…1:640
5	44	F	DLE + mucosal lesions	HCQ, stopped: belimumab			1:320
6	37	F	DLE	MTX	5	0	Negative
7	22	M	DLE + mucosal lesions	HCQ, MMF, stopped: baricitinib			1:1,280

**Figure 1:**
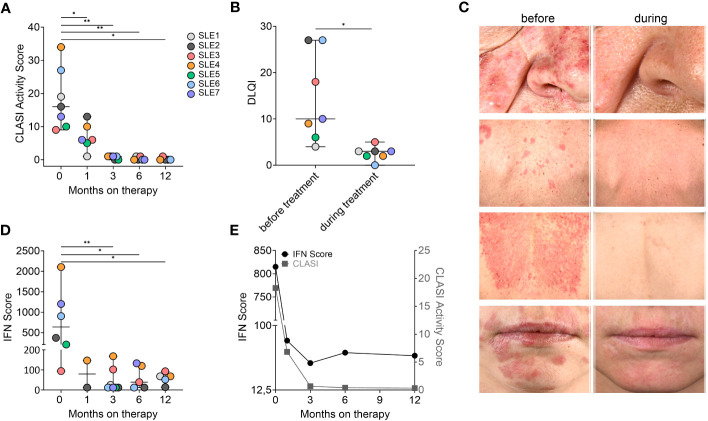
Rapid response to anifrolumab in patients with CLE. **(A)** CLASI-A scores in patients with SLE before and during anifrolumab treatment. **(B)** DLQI during treatment. **(C)** SCLE and CDLE lesions rapidly responding to anifrolumab treatment. **(D)** IFN score during treatment. qRT-PCR of PBMCs was performed to measure the expression of seven individual genes (IFI27, IFI44, IFI44L, IFIT1, ISG15, SIGLEC1, and RSAD2) normalized to GAPDH and HPRT1 and compared to a cohort of healthy controls. The score is calculated as previously described (2). A negative score is defined as <12.49 (mean ISG score of 10 controls plus two SD), and graphs show mean and SD. Unpaired Student’s *t*-test, **p < 0.01, *p < 0.05. **(E)** Mean decline of IFN and CLASI scores in all patients over time.

The colours correspond to the colours in **Figure 1**. This allows to identify the clinical data for each patients 1-7 in the **Figure 1**.

IFN scores were determined by measuring the mRNA expression of seven IFN-stimulated genes (IFI27, IFI44, IFI44L, IFIT1, ISG15, SIGLEC1, and RSAD2) normalized to GAPDH and HPRT1 and compared to a healthy cohort in peripheral blood mononuclear cells.

## Results

A rapid clinical response beginning with the first infusion was reported by all patients. Previously refractory skin lesions were improved by 50% after 1 month as measured by median CLASI - activity (CLASI-A) and nearly completely resolved within 3–12 months of treatment (**Figures 1A, C**). Importantly, this clinical response was seen in all subtypes of cutaneous lupus and even chronic discoid lesions rapidly responded to treatment (**Figure 1C**, **Table 1**). Mucosal lesions in patients 4, 5, and 7 responded as rapidly as cutaneous lesions to anifrolumab treatment and did not relapse during the observation period. No patients had significant alopecia. Relief of cutaneous symptoms was associated with improvement in quality of life (**Figure 1B**). The patients did not report significant adverse events. One patient experienced a prolonged respiratory tract infection. However, this was not followed by SLE exacerbation. A 23-year-old patient also reported mild viral respiratory infections and herpes zoster. In contrast to nearly complete resolution of cutaneous symptoms, ANA were still detectable in sera of patients and declined only in one individual (**Table 1**). In addition, we did not observe a normalization of complement levels in patients (data not shown). However, the general severity of the disease was reduced, and one patient could taper prednisolone, which she frequently requested for monthly flares, from 20 mg to 5 mg per day (**Table 1**).

In parallel to the clinical response, we observed a reduction in IFN scores in blood (**Figures 1D, E**). IFN scores continued to decline over 6 to 12 months of treatment. Our data reveal that IFN scores in contrast to CLASI-A did not completely resolve during therapy in most of the patients (**Figure 1E**).

## Discussion

The clinical response to anifrolumab is remarkable and underscores the dependence of cutaneous lupus on type I IFN signaling. It further substantiates previously reported observations in the treatment of refractory SLE patients with CLE (3–5). The incomplete resolution of IFN scores might indicate that the cutaneous response is not entirely dependent on systemic reduction of IFN-stimulated genes (ISG) in blood. On the other hand, the observed induction of these genes could potentially result from other pathways, e.g., IFN-gamma.

In addition, the analysis of Carter et al. demonstrated that only a certain subset of ISGs responds to anifrolumab treatment whereas others remain unchanged (4). The selection of ISGs is therefore critical for the development of the IFN signature as biomarker in CLE and SLE. The ISG subset chosen here was based on previous data demonstrating their upregulation in interferonopathies and the response to anti-inflammatory treatment in monogenic, type I IFN-driven forms of LE (6). All selected seven ISGs decreased during treatment. Interestingly, our cohort included two patients with a known genetic predisposition to SLE based on C1 or RNaseH2B mutations, indicating that the response to anifrolumab is as affective in monogenic as multifactorial forms of SLE (2, 7).

In conclusion, these findings demonstrate impressive clinical response to anifrolumab among different subsets of CLE in SLE patients previously refractory to treatment and suggest that a certain subset of ISGs could be implemented as a valuable biomarker in CLE.

## Data availability statement

The raw data supporting the conclusions of this article will be made available by the authors, without undue reservation.

## Ethics statement

The studies involving humans were approved by ethics committee University Dresden, Germany. The studies were conducted in accordance with the local legislation and institutional requirements. The participants provided their written informed consent to participate in this study. Written informed consent was obtained from the individual(s) for the publication of any potentially identifiable images or data included in this article.

## Author contributions

CG: designing research studies, writing manuscript, acquiring patients. LF, CW, and SR: conducting experiments and analysing data. SB discussing data and patients. MA: acquiring patients, writing manuscript. ML-K: analysing data, writing manuscript. All authors contributed to the article and approved the submitted version.
